# Effect of genetic and environmental influences on cardiometabolic risk factors: a twin study

**DOI:** 10.1186/1475-2840-10-96

**Published:** 2011-11-03

**Authors:** György Jermendy, Tamás Horváth, Levente Littvay, Rita Steinbach, Ádám L Jermendy, Ádám D Tárnoki, Dávid L Tárnoki, Júlia Métneki, János Osztovits

**Affiliations:** 1 Medical Department, Bajcsy-Zsilinszky Hospital, Maglódi út 89-91, Budapest, 1106 Hungary; 2 Institute of Human Physiology and Clinical Experimental Research, Semmelweis University, Tűzoltó út 37-47, Budapest, 1094 Hungary; 3 Central European University, Nádor út 9, Budapest, 1051 Hungary; 4 Semmelweis University, Faculty of Medicine, Üllői út 26, Budapest, 1085 Hungary; 5 Department of Radiology and Oncotherapy, Semmelweis University, Üllői út 78/a, Budapest, 1082 Hungary; 6 National Centre for Healthcare Audit and Inspection, Gyáli út 2-6, Budapest, 1097 Hungary

**Keywords:** cardiometabolic risk, diabetes mellitus, cardiovascular diseases, twin study, heritability, cardiovascular prevention

## Abstract

**Background:**

Both genetic and environmental factors play a role in the pathogenesis of type 2 diabetes and cardiovascular diseases. The magnitude of genetic and environmental influences may vary in different populations and can be investigated by twin studies.

**Methods:**

In this cross-sectional study, 101 (63 monozygotic and 38 dizygotic) adult twin pairs (n = 202; mean age: 44.3 ± 15.8 years) were investigated. Past medical history was recorded and physical examination was performed. Fasting venous blood samples were taken for measuring laboratory parameters. For assessing heritability of 14 cardiovascular risk factors, the structural equation (A-C-E) model was used.

**Results:**

The following risk factors were highly (> 70.0%) or moderately (50.0 - 69.0%) heritable: weight (88.1%), waist circumference (71.0%), systolic blood pressure (57.1%), diastolic blood pressure (57.7%), serum creatinine (64.1%), fibrinogen (59.9%), and serum C-reactive protein (51.9%). On the other hand, shared and unique environmental influences had the highest proportion of total phenotypic variance in serum total cholesterol (46.8% and 53.2%), serum HDL-cholesterol (58.1% and 14.9%), triglycerides (0.0% and 55.9%), fasting blood glucose (57.1% and 42.9%), fasting insulin (45.4% and 54.5%), serum uric acid (46.0% and 31.3%), and serum homocysteine (71.8% and 28.2%, respectively).

**Conclusion:**

Some cardiometabolic risk factors have strong heritability while others are substantially influenced by environmental factors. Understanding the special heritability characteristics of a particular risk factor can substantiate further investigations, especially in molecular genetics. Moreover, identifying genetic and environmental contribution to certain cardiometabolic risk factors can help in designing prevention and treatment strategies in the population investigated.

## Background

The term of cardiometabolic risk designates the global risk for diabetes mellitus and cardiovascular diseases. Beyond age, race, sex and family history, other classic and newly recognized risk factors such as abnormal lipid parameters, obesity, insulin resistance syndrome, smoking, hypertension, hypercoagulation and inflammation are globally covered by the term of cardiometabolic risk [1]. Recently, the clinical usefulness of the metabolic syndrome became questionable and instead of its use the evaluation of global cardiometabolic risk has been widely recommended [2,3].

Cardiometabolic risks in adulthood differ due to several factors such as familial background, ethnicity, geographical areas, people's life style and socioeconomic situation [4]. Shortly, they are influenced by genetic and environmental factors. As a result, the prevalence rate of cardiovascular diseases and type 2 diabetes mellitus may vary in different populations. Unfortunately, people living in Central European region are at increased risk for cardiovascular morbidity and mortality compared to other European nations [5] while the prevalence rate of type 2 diabetes is in line with other European countries [6].

Genetic and environmental influences on cardiometabolic risk factors can be investigated by twin studies. Obviously, understanding the influences of genetic and environmental factors would provide insights into the pathomechanism of type 2 diabetes and cardiovascular diseases. Moreover, the effectiveness of different treatments might be anticipated more correctly knowing the influence of genetic and environmental factors on cardiometabolic risk factors.

In our classical twin study, 14 different risk factors were simultaneously assessed in monozygotic (MZ) and dizygotic (DZ) adult twin pairs without diabetes and known cardiovascular diseases in order to determine the genetic and environmental influences on cardiometabolic risk factors.

## Methods

### Patient cohort

In this cross-sectional twin study, 101 adult twin pairs (n = 202; women 72.3%; mean age 44.3 years, range 18-81 years) were investigated. As no population based twin registry is available in Hungary at present, participants were recruited from national twin meetings and through advertisements published in local newspapers. Exclusion criteria included pregnancy, diabetes mellitus, myocardial infarction or regular alcohol consumption (more than 2 units daily) in the past medical history and acute infection within three weeks of measurement.

Anthropometric parameters (weight, height, waist circumference) were recorded and a complete physical examination including blood pressure measurement in sitting position was performed. Body mass index (BMI) was calculated from the values of weight and height. Waist circumference was measured conventionally [7]. Physical activity level was assessed by the standardized method: subjects reported the amount of time spent on five different intensity levels of physical activity on an average weekday as a total 24 h, then values of daily metabolic equivalent score (MET) were derived and used for statistical analysis [8]. Smoking habit was assessed as smoking years while alcohol consumption was evaluated as unit per week. Fasting venous blood samples were taken from twin pairs and routine laboratory methods were used for measuring laboratory parameters. The value of homeostasis model assessment - insulin resistance (HOMA-IR) was calculated using the widely accepted formula [9]: HOMA-IR = fasting blood glucose (mmol/l) × fasting insulin (μU/ml)/22.5.

Due to the lack of genotyping data of subjects, we used a multiple self-reported question approach to assess zygosity in order to maximize the accuracy of classification. The most likely zygosity was assigned based on the seven self-reported responses [10]. In this way, 63 MZ and 38 DZ twin pairs were investigated.

All participants provided informed consent. The investigation was approved by the National Research Ethics Committee (ETT TUKEB, Budapest) and was conducted according to the principles expressed in the Declaration of Helsinki. Participants were informed about the results of the investigation and medical advice was provided when needed.

### Statistics

Descriptive statistical analysis was used for characterizing MZ and DZ twin pairs. The differences in clinical and laboratory findings between MZ and DZ twin pairs were evaluated by using Mann-Whitney test. The level of significance was set at p < 0.05.

Bivariate correlation between the same age- and gender-corrected parameters was separately analyzed in MZ and DZ pairs. If coefficients of correlations (r values) were high in MZ and not in DZ pairs, a genetic effect could be assumed. On the other hand, if r values were lower or close to each other when MZ and DZ pairs were compared, an environmental effect could be supposed.

For assessing heritability, a structural equation model, often called the A-C-E model, was used. In this model, three latent variables, additive genetic effects ("A"), common (or shared) environment ("C") and unshared (or unique) environment ("E") drive the variance in the phenotype for each twin [11]. "A" is perfectly (1.0) correlated across MZ twins and 0.5 correlated across DZ twins. "C" is perfectly correlated independently of zygosity. "E" is uncorrelated across co-twins. Since measurement error in the phenotype is also uncorrelated across measurements, it appears as part of the unique environmental component. Considering the well established, reliable measures used in this study this property of the model is of little concern. Empirically derived bootstrapped confidence intervals are presented for the heritability and environmental proportion estimates [12]. All inferential statistics were estimated using full information maximum likelihood with the software Mplus Version 6 [13].

For each phenotype two A-C-E models were estimated. Model-1 corrects for the twins' age and gender. The correction for age and gender is justified by gender being 100% genetic and age being 100% environmental. All other predictors could carry both a genetic and an environmental component. Results from Model-1 tell us the total genetic and environmental impact on the dependent variable. Model-2, in addition to age and gender, also corrects for BMI and waist circumference. Making a comparison of results in Model-1 and Model-2 analysis, effect of BMI and waist circumference on the phenotypic variance could be evaluated.

Based on the results of A-C-E model, genetic influence was considered high if > 70% of the total variance was related to additive genetic factors ("A"). Genetic influence was designated as moderate if additive genetic factors ("A") contributed between 50.0% and 69.0% to the total variance of phenotype measured. If genetic influence was < 50.0% environmental factors have substantial impact on the phenotypic variance.

Heritability of calculated parameters (BMI, HOMA-IR, estimated glomerular filtration rate [eGFR]) was not investigated. Instead, heritability of their components (weight, height, fasting blood glucose, fasting insulin, serum creatinine) was analyzed only.

## Results

Although a significant difference between ages of MZ versus DZ twin pairs occurred, there were no significant differences between gender, anthropometric parameters, smoking and drinking habits as well as physical activity. As for other cardiovascular risk factors, systolic blood pressure, serum total cholesterol and triglycerides values were significantly higher in MZ versus DZ twin pairs. No significant differences were found between diastolic blood pressure and other laboratory parameters when MZ and DZ twins were compared (Table 1.)

**Table 1:** Clinical and laboratory findings of 63 monozygotic (MZ) and 38 dizygotic (DZ) twin pairs (x ± SD or %)

	MZ twins (n = 126)	DZ twins (n = 76)	p value
**Women (%)**	73.0	71.1	0.832
**Age (years)**	47.4 ± 15.5	38.3 ± 13.5	< 0.001
**Duration of smoking (years)**	4.8 ± 9.5	4.8 ± 9.6	0.829
**Alcohol consumption (unit/week)**	1.1 ± 2.2	1.9 ± 3.4	0.553
**Physical activity (MET/24 hours)**	65.8 ± 22.2	60.3 ± 21.1	0.094
**Weight (kg)**	71.1 ± 14.5	72.4 ± 17.6	0.827
**Height (cm)**	165 ± 9	167 ± 9	0.211
**Body mass index (kg/m^2^)**	25.9 ± 4.9	25.8 ± 5.9	0.576
**Waist circumference (cm)**	88 ± 14	88 ± 16	0.757
**Systolic blood pressure (mmHg)**	130.2 ± 14.8	125.3 ± 14.1	0.026
**Diastolic blood pressure (mmHg)**	74.7 ± 10.3	72.6 ± 9.8	0.152
**Serum total cholesterol (mmol/l)**	5.35 ± 1.23	5.00 ± 1.07	0.031
**Serum HDL-cholesterol (mmol/l)**	1.60 ± 0.39	1.60 ± 0.36	0.755
**Serum triglycerides (mmol/l)**	1.33 ± 0.86	1.07 ± 0.80	0.004
**Fasting blood glucose (mmol/l)**	5.01 ± 0.75	4.81 ± 0.63	0.064
**Fasting insulin (μU/ml)**	7.35 ± 5.15	6.83 ± 4.64	0.665
**HOMA-IR**	1.70 ± 1.39	1.53 ± 1.29	0.404
**Serum creatinine (μmol/l)**	70.0 ± 9.8	72.3 ± 11.4	0.219
**Serum uric acid (μmol/l)**	286.3 ± 75.3	282.2 ± 74.8	0.584
**Fibrinogen (g/l)**	3.27 ± 0.69	3.18 ± 0.72	0.219
**Serum homocysteine (μmol/l)**	12.1 ± 5.64	10.9 ± 2.9	0.408
**Serum C-reactive protein (mg/l)**	3.82 ± 6.53	4.27 ± 5.01	0.871

MET: metabolic equivalent score

HOMA-IR: homeostatis model assessment insulin resistance

Assessing bivariate correlation between age- and gender-corrected parameters measured separately in MZ and DZ twin pairs, the coefficients of correlation (r values) were high for anthropometric parameters, blood pressure, serum creatinine, fibrinogen and high sensitivity C-reactive protein (hsCRP) values in MZ twins whereas those for the same measurements were much lower in DZ twins. As for serum total cholesterol, triglycerides, HDL-cholesterol, fasting blood glucose, fasting insulin, serum uric acid and homocysteine values, the co-twin correlation was not so strong or r values were very close to each other for both zygosity groups (Table 2).

**Table 2:** Bivariate correlation (r values and 95% CI) between age- and gender-corrected parameters measured in monozygotic (MZ) and dizygotic (DZ) twin pairs

	MZ twins (n = 126; 63 twin pairs)		DZ twins (n = 76; 38 twin pairs)	
	
	r value	95% CI	r value	95% CI
**Weight**	0.881	0.800-0.931	0.437	0.077-0.737
**Waist circumference**	0.865	0.763-0.935	0.510	0.052-0.781
**Systolic blood pressure**	0.598	0.426-0.731	0.055	-0.524-0.576
**Diastolic blood pressure**	0.608	0.444-0.728	0.147	-0.321-0.648
**Serum total cholesterol**	0.445	0.259-0.633	0.640	0.363-0.845
**Serum HDL-cholesterol**	0.840	0.626-0.895	0.658	-0.293-0.798
**Serum triglycerides**	0.527	0.281-0.707	0.349	-0.023-0.625
**Fasting blood glucose**	0.523	0.453-0.804	0.545	0.068-0.744
**Fasting insulin**	0.513	0.292-0.725	0.492	0.209-0.716
**Serum creatinine**	0.639	0.479-0.784	0.159	-0.065-0.434
**Serum uric acid**	0.691	0.542-0.799	0.629	0.453-0.772
**Fibrinogen**	0.663	0.499-0.791	0.322	0.024-0.639
**Serum homocysteine**	0.681	0.505-0.781	0.838	0.582-0.948
**Serum C-reactive protein**	0.683	0.460-0.851	0.138	-0.199-0.555

**Figure 1:**
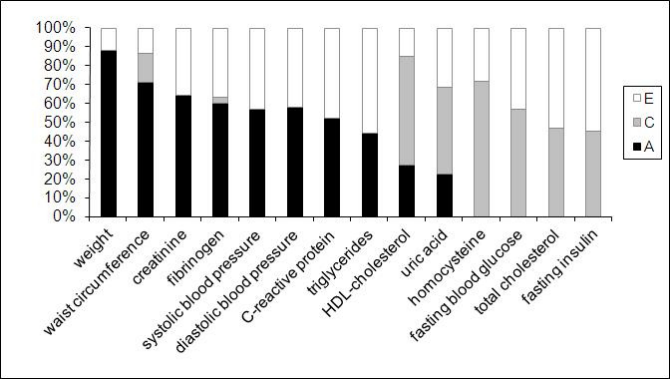
**Mean values of additive genetic (A), common (C) and unique environmental (E) influences on cardiometabolic risk factors (univariate structural equation model [A-C-E] analysis in 101 twin pairs)**. Weight and waist circumference are corrected for age and gender. All other values are corrected for age, gender, waist circumference and BMI.

Using the structural equation model, the following risk factors were highly or moderately heritable: weight (88.1%; 95% CI: 66.0-94.0), waist circumference (71.0%; 95% CI: 17.7-91.8), systolic blood pressure (57.1%; 95% CI: 27.4-73.5), diastolic blood pressure (57.7%; 95% CI: 23.7-74.4), serum creatinine (64.1%; 95% CI: 34.6-78.5), fibrinogen (59.9%; 95% CI: 20.5-78.5), and serum hsCRP (51.9%; 95% CI: 0.0-79.4). On the other hand, shared and unique environmental influences had the highest proportion of total phenotypic variance in serum total cholesterol (46.8 and 53.2%), serum HDL-cholesterol (58.1 and 14.9%), triglycerides (0.0% and 55.9%), fasting blood glucose (57.1 and 42.9%), fasting insulin (45.4 and 54.5%), serum uric acid (46.0 and 31.3%), and serum homocysteine (71.8 and 28.2%, respectively) (Figure 1). Comparing the results of the initial and final analysis (Model-1 and Model-2), the difference in A-C-E model varied from 0.0% to 17.1%, indicating that only a minor proportion of the influences can be explained by the effects of anthropometric parameters (Table 3).

**Table 3:** Parameter estimates for additive genetics (A), common environmental (C) and unique environmental (E) influences on cardiometabolic risk factors (A-C-E univariate model analysis)

		Parameter estimates		
		
		„A" Additive genetics (95% CI)	„C" Common environmental (95% CI)	„E" Unique environmental (95% CI)
**Weight**	**Model-1**	88.1% (66.0 - 94.0)	0.0% (0.0 - 56.8)	11.9% (6.9 - 20.1)
**Waist circumference**	**Model-1**	71.0% (17.7 - 91.8)	15.5% (0.0 - 68.0)	13.5% (6.5 - 23.9)
**Systolic blood pressure**	**Model-l**	59.0% (35.6 - 74.1)	0.0% (0.0 - 0.0)	41.0% (26.5 - 58.2)
	**Model-2**	57.1% (27.4 - 73.5)	0.0% (0.0 - 65.9)	42.9% (28.9 - 61.1)
**Diastolic blood pressure**	**Model-1**	60.4% (25.0 - 75.0)	0.0% (0.0 - 36.2)	39.6% (26.5 - 55.8)
	**Model-2**	57.7% (23.7 - 74.4)	0.0% (0.0 - 65.6)	42.3% (27.8 - 60.2)
**Serum total cholesterol**	**Model-1**	0.0% (0.0 - 65.5)	50.1% (35.6 - 65.5)	49.9% (33.7 - 64.8)
	**Model-2**	0.0% (0.0 - 70.2)	46.8% (30.4 - 64.2)	53.2% (35.8 - 69.3)
**Serum HDL-cholesterol**	**Model-1**	36.4% (3.9 - 69.5)	47.6% (14.9 - 71.9)	16.0% (9.4 - 23.1)
	**Model-2**	27.1% (0.0 - 62.1)	58.1% (25.1 - 81.8)	14.9% (8.3 - 23.0)
**Serum triglycerides**	**Model-1**	35.6% (0.0 - 68.6)	17.1% (0.0 - 56.7)	47.3% (28.9 - 66.9)
	**Model-2**	44.1% (22.2 - 65.1)	0.0% (0.0 - 0.0)	55.9% (34.8 - 77.6)
**Fasting blood glucose**	**Model-1**	0.0% (0.0 - 57.8)	53.1% (34.8 - 69.3)	46.9% (33.6 - 61.2)
	**Model-2**	0.0% (0.0 - 34.4)	57.1% (36.4 - 74.8)	42.9% (28.4 - 59.8)
**Fasting insulin**	**Model-1**	4.2% (0.0 - 60.4)	47.1% (18.1 - 69.0)	48.7% (30.1 - 67.8)
	**Model-2**	0.1% (0.0 - 62.8)	45.4% (13.6 - 68.1)	54.5% (34.2 - 77.6)
**Serum creatinine**	**Model-1**	62.3% (44.9 - 78.4)	0.0% (0.0 - 0.0)	37.7% (21.6 - 53.9)
	**Model-2**	64.1% (34.6 - 78.5)	0.0% (0.0 - 56.2)	35.9% (22.2 - 51.8)
**Serum uric acid**	**Model-1**	12.4% (0.0 - 48.1)	56.7% (25.7 - 74.2)	30.9% (20.5 - 45.8)
	**Model-2**	22.7% (0.0 - 74.5)	46.0% (0.0 - 71.8)	31.3% (18.6 - 46.1)
**Fibrinogen**	**Model-1**	66.2% (41.1 - 83.5)	0.0% (0.0 - 25.1)	33.8% (20.8 - 50.3)
	**Model-2**	59.9% (20.5 - 78.5)	3.4% (0.0 - 57.7)	36.7% (23.0 - 57.3)
**Serum homocysteine**	**Model-1**	0.0% (0.0 - 69.9)	72.4% (53.7 - 80.6)	27.6% (19.4 - 45.1)
	**Model-2**	0.0% (0.0 - 46.9)	71.8% (50.5 - 79.7)	28.2% (20.3 - 47.9)
**Serum C-reactive protein**	**Model-1**	66.0% (26.0 - 87.0)	0.0% (0.0 - 43.4)	34.0% (15.0 - 59.1)
	**Model-2**	51.9% (0.0 - 79.4)	0.0% (0.0 - 52.4)	48.1% (20.7 - 81.6)

Model-1: values corrected for age and gender

Model-2: values corrected for age, gender, waist circumference and body mass index

## Discussion

Several cardiometabolic risk factors (weight, waist circumference, blood pressure, serum creatinine, fibrinogen and serum hsCRP) were highly or moderately heritable in our Hungarian twin cohort while others (serum total cholesterol, HDL-cholesterol, triglycerides, fasting blood glucose, fasting insulin, serum uric acid and homocysteine) were substantially influenced by shared and unique environmental factors.

The pathomechanism of atherosclerosis is a complex process involving several traditional and novel cardiovascular risk factors. In this respect, both genetic and environmental factors can be identified. Recently, the term of cardiometabolic risk is widely used for risk factors contributing to both cardiovascular and metabolic diseases [1]. Clearly, cardiovascular diseases and type 2 diabetes are believed to be multifactorial in origin indicating that many genes alone or in combination with environmental factors contribute to the development of the phenotypic variance. Classical twin studies, such as ours, can help to estimate the genetic and environmental components of variance of the traits.

It is well documented that beyond weight (BMI), waist circumference, blood pressure, serum lipids and blood glucose [14,15], serum creatinine (eGFR) [16], serum uric acid [17], fibrinogen [18], homocysteine [19] and hsCRP [20] could also be considered as cardiovascular or cardiometabolic risk factors. Fasting insulin could be a sign of hyperinsulinaemia or insulin resistance and may contribute to cardiometabolic risk [21]. In contrast to others [15], heritability of BMI, HOMA-IR, eGFR and that of the metabolic syndrome was not assessed in our study because heritability of calculated values has no or limited biological relevance. As for the metabolic syndrome, its clinical usefulness is widely debated and the term is considered rather an educational concept than a practical tool in the diagnosis and treatment [2,3]. Therefore, heritability of the metabolic syndrome components was analyzed only in our study.

The strong heritability of anthropometric parameters (weight, waist circumference) in our cohort is in line with former investigations [22]. Moreover, the high genetic determination of systolic and diastolic blood pressure values in our study corresponds to other clinical observation [23]. As for lipid parameters, heritability varied between 8-72% for total cholesterol, 21-79% for HDL-cholesterol and 19-72% for serum triglycerides in different studies [15]. Our results (heritability < 50%) corroborate the importance of environmental influences. The environmental determination of fasting blood glucose and fasting insulin in our study is in accordance with observation of others [15].

Several clinical studies documented that cardiovascular morbidity and mortality are associated with chronic kidney diseases characterized by elevated serum creatinine and decreasing eGFR values [16]. In our study, genetic factors had moderate influence (64.1%) on serum creatinine value. This is in line with results of other twin or family studies [24-26].

Serum uric acid should be considered as an independent cardiovascular risk factor [17]. No dominant genetic influence (22.7%) on serum uric acid values was found in our cohort. In a recent study [27], genetic determination of serum uric acid values was found to be 42% while this number varied from 40% to 73% in other studies reviewed.

The level of fibrinogen can reflect to hypercoagulation and is considered among cardiovascular risk factors [18]. The moderate genetic influence (59.9%) on phenotypic variance of fibrinogen in our study is in line with earlier observations [28].

Homocysteine is listed among the relatively newer cardiovascular risk factors [19], although the results of interventional studies proved to be disappointing [29]. In our study, the value of serum homocysteine was influenced by both shared and unique environmental factors (71.8% and 28.2%, respectively). This observation corroborates the findings from *Cesari et al *[30]. It is of note, however, that strong heritability was reported by others [31,32].

The value of hsCRP is a marker of low grade inflammation and is recently considered as a cardiovascular risk factor both in epidemiological and interventional studies [18]. A moderate heritability (51.9%) in hsCRP value was found in our twin cohort, similarly to others [33].

Taken our results together, it is obvious that some minor discrepancies in numerical genetic and environmental influences on cardiometabolic risk factors exist when our results are compared to those of formerly performed twin studies. However, it is not a surprise because different populations were investigated. It is of note, therefore, that our results should be considered valid only in Hungarian adult people without diabetes and cardiovascular diseases.

Beyond classical twin studies [34], the pathomechanism, especially the genetic susceptibility to cardiovascular diseases, could also be investigated by methods of molecular genetics [35]. Recently, some aspects of cardiometabolic disease heritability were highlighted by gene association studies. This was the case for type 2 diabetes and the metabolic syndrome [36-38] but functional gene variants were observed also in hypertension [39] and in lipid abnormalities [40]. More importantly, developments in molecular genetics and pharmacogenetics have already resulted in better understanding of the underlying pathomechanism of certain cardiometabolic diseases providing the possibility of disease prevention or individualized therapies for affected patients in the future [41].

If a cardiometabolic risk factor proved to be highly or moderately heritable it does not mean that the phenotype cannot be influenced. Rather, early and sustained intervention is needed. Clearly, if a risk factor has strong heritability, beneficial effect of any intervention can only be successful if it is implemented in early adulthood or, even better, in early childhood. In addition, recent progress in molecular genetics and pharmacogenetics holds the promise of individualized therapies for patients carrying disease predisposing genotypes [42]. On the contrary, it can be assumed that a risk factor with substantial environmental determination can much easily be affected by changes in environments. Nevertheless, the individual variation in the result of intervention may be high.

Our study has some limitations. The sample size was modest but comparable to other clinical studies with twins [28]. The zygosity in our twin cohort was classified according to validated questionnaires. Nevertheless, this method is widely accepted in clinically oriented twin studies [10]. Our results were derived from a healthy adult twin population, therefore, the extrapolation of our findings to patients with clinically manifest cardiovascular diseases or diabetes has some limitations. Although diabetes mellitus and myocardial infarction in the past medical history were considered as exclusion criteria, oral glucose tolerance test and detailed cardiological investigations were not carried out in our cohort.

The strengths of our study are worth mentioning. All subjects were investigated by the same investigators (JO, RS, ÁLJ, ÁDT, DLT) in one department (Bajcsy-Zsilinszky Hospital). All laboratory parameters were centrally measured in the same hospital immediately after taking venous blood sample. In our study, 14 cardiometabolic risk factors were simultaneously evaluated. The number of twin studies evaluating heritability of different cardiometabolic risk factors in such a large scale is limited [15,43]. To the best of our knowledge this is the first twin study from Hungary evaluating heritability of different cardiometabolic risk factors.

## Conclusions

Our study indicated that the influence of genetic and environmental factors on cardiometabolic risk factors' phenotype can be varied within a broad range. Some of them have strong heritability while others are substantially influenced by environmental factors. The better understanding of genetic and environmental contribution to certain cardiometabolic risk factors can help in designing prevention and treatment strategies in the population investigated.

## List of abbreviations

A-C-E model: A: additive genetics, C: common (shared) environmental, E: unique (unshared) environmental estimates; BMI: body mass index; DZ: dizygotic; eGFR: estimated glomerular filtration rate; HOMA-IR: homeostasis model assessment - insulin resistance; hsCRP: high sensitivity C-reactive protein; MET: metabolic equivalent score; MZ: monozygotic.

## Competing interests

The authors declare that they have no competing interests.

## Authors' contributions

GJ conceived of the study, participated in its designs and coordination and wrote the final manuscript. TH, RS, ÁLJ, ÁDT, TLD and JM participated in clinical investigations and data collection, have been involved in drafting the manuscript. LL participated in the design of the study, performed the statistical analysis and has been involved in drafting the manuscript. JO participated in study design and coordination, performed clinical investigation and data collection, and helped to draft the manuscript. All authors read and approved the final manuscript.
